# Identification of Key Metabolites for Acute Lung Injury in Patients with Sepsis

**Published:** 2019-01

**Authors:** Pei-Quan WANG, Jing LI, Li-Li ZHANG, Hong-Chun LV, Su-Hua ZHANG

**Affiliations:** 1. Department of Intensive Care Unit, Linzi District People’s Hospital, Zibo, Shandong 255400, China; 2. Department of Geratology, Linzi District People’s Hospital, Zibo, Shandong 255400, China; 3. Department of Health Care, Qilu Hospital of Shandong University (Qingdao), Qingdao, Shandong 266000, China

**Keywords:** Acute lung injury, Metabolites, Multi-omics network, Differentially expressed genes

## Abstract

**Background:**

The study aimed to detect critical metabolites in acute lung injury (ALI).

**Methods:**

A comparative analysis of microarray profile of patients with sepsis-induced ALI compared with sepsis patients with was conducted using bioinformatic tools through constructing multi-omics network. Multi-omics composite networks (gene network, metabolite network, phenotype network, gene-metabolite association network, phenotype-gene association network, and phenotype-metabolite association network) were constructed, following by integration of these composite networks to establish a heterogeneous network. Next, seed genes, and ALI phenotype were mapped into the heterogeneous network to further obtain a weighted composite network. Random walk with restart (RWR) was used for the weighted composite network to extract and prioritize the metabolites. On the basis of the distance proximity among metabolites, the top 50 metabolites with the highest proximity were identified, and the top 100 co-expressed genes interacted with the top 50 metabolites were also screened out.

**Results:**

Totally, there were 9363 nodes and 10,226,148 edges in the integrated composite network. There were 4 metabolites with the scores > 0.009, including CHITIN, Tretinoin, sodium ion, and Celebrex. Adenosine 5′-diphosphate, triphosadenine, and tretinoin had higher degrees in the composite network and the co-expressed network.

**Conclusion:**

Adenosine 5′-diphosphate, triphosadenine, and tretinoin may be potential biomarkers for diagnosis and treatment of ALI.

## Introduction

Acute lung injury (ALI) is a severe systemic inflammatory response syndrome characterized by refractory hypoxemia and respiratory distress, resulting in respiratory failure and subsequent mortality (1). Common causes of ALI include serious infection, sepsis, aspiration, shock, as well as trauma (2), and sepsis is the most common one (3). Worriedly, the mortality is approximately 40% and the survival rate in patients with significant lung injury is about 50% (4). Thus, it is a major challenge to elucidate the molecular mechanisms of sepsis-induced ALI, and to early diagnose ALI patients.

As we all know, the present clinical standard for diagnosing ALI covers hypoxemia, pulmonary edema, as well as capillary leakage. Of note, an unconformity exists between clinical standard and histological autopsy results (5). Due to this difficulty in diagnosis, it is urgently imperative to detect biomarkers for ALI. Fortunately, gene expression analysis has been performed, microarray technology enhances extraction of diagnostic and prognostic biomarkers, and sheds insights on the pathology of disease. For example, GSE10474, developed by Howrylak in 2009 (6), is a microarray data about ALI, in which, an eight-gene expression profile that can distinguish between patients with ALI plus sepsis and sepsis patients accurately. Moreover, Chen et al. (7) have used the same expression profile (6) to further discover 12 differentially expressed genes (DEGs) and 7 important functions. Guo et al. (8) also used this microarray data to identify several key genes (PTK2, SRC and CAV2) for ALI in patients with sepsis. Although these studies are helpful to reveal the molecular mechanism of ALI, the DEGs obtained in these studies were not consistent. In addition, the metabolites of ALI have not studied so far. As demonstrate, extracting the disease-related metabolites is highly important for improving clinical diagnosis, and for a better understanding of metabolic pathological processes (9, 10).

With the aim of detecting the novel and significant metabolites for ALI, we used the same microarray expression data of ALI (GSE10474) to compare the chip data from septic ALI with those from sepsis patient to uncover DEGs. Moreover, a weighted composite network was constructed through integrating six data set (gene network, metabolite network, phenotype network, gene-metabolite association network, phenotype-gene association network, and phenotypemetabolite association network). Then, candidate metabolite prioritization was implemented based on the weighted composite network. We believe that our study will provide several insights of the understanding of etiology on how ALI initiates and progresses. More significantly, candidate metabolites might offer the basis for the early detection and treatment for ALI.

## Materials and Methods

### Gene expression data

Expression profile of GSE10474 (6) was obtained from gene expression omnibus database (GEO, http://www.ncbi.nlm.nih.gov/geo/), which was determined using the platform of Affymetrix Human Genome U133A 2.0 Array (Affymetrix Inc., Santa Clara, California, USA). In GSE10474, there were 13 whole blood samples with ALI plus sepsis and 21 whole blood samples in patients with sepsis alone. We downloaded the raw data for subsequent analysis.

### Pre-treatment and differential analysis

Raw probes were read with package affy of R (11). Then, robust multichip averaging (RMA) method was used to conduct pre-treatment including background adjustment, normalization and expression value calculation. After the probes were aligned to the gene symbols, we obtained a total of 11,199 genes. Differential analysis was performed based upon t-test. The adjusted p-value (FDR) < 0.05 was selected as the cutoff criteria. Importantly, these DEGs were used to construct the following gene-gene network.

### Construction of multi-omics composite network

A composite network was established through integrating 6 data, which were denoted by 6 networks (gene network, metabolite network, phenotype network, gene-metabolite network, phenotype-gene network, and phenotype-metabolite network). Next, we would looked at the detailed description.

### Gene network

In this analysis, we downloaded all human protein-protein interactions (PPIs) having combine-scores (1,048,576 interactions) from the STRING database to further construct the background PPI network. After eliminating the duplicated PPIs, and converting proteins into genes, 1,515,370 highly correlated gene interactions (covering 16,785 genes) were extracted to build the seed PPI network (herein, combine-score of interactions not less than 0.8). Then, we took the intersection between the 16,785 genes of the seed PPI network and DEGs to establish the informative gene-gene network.

### Metabolite network

There were 4,994 human metabolites, which were gathered from the human pathways of Reactome, MSEA (12) and SMPDB (13), and from the metabolite pathways of KEGG and HMDB. Subsequently, metabolite-metabolite interactions of human and the corresponding confidence scores were collected from STITCH (14), in which the metabolites must be included in the 4,994 human metabolites. At the end, we acquired 3,764 human metabolites and 74,667 human metabolite associations (not all metabolites interacted in STITCH).

### Phenotype network

As demonstrated, 5,080 phenotypes and the similarity scores across them were covered in the phenotype-phenotype interactions (15), The majority of documented phenotypes in human were included in these phenotypes. On the basis of the phenotype-phenotype similarity interactions, a phenotype network was constructed.

### Gene-metabolite association network

To obtain the gene-metabolite associations in human, we collected the human chemical and gene interactions and their confidence scores from the STITCH. Based on the 4,994 human metabolites, human metabolite and gene associations were obtained. After getting rid of the metabolites not included in the metabolite network and eliminating the genes not involved in the gene network, 192,763 gene-metabolite interactions were obtained (covering 12,342 genes, and 3,278 metabolites).

### Phenotype-gene association network

To begin with, the phenotype-gene interactions were obtained relying on the curated Morbid Map file of the OMIM database. When discarding the phenotypes that were not included in the phenotype network and the genes that were not involved in the gene network, 1,715 genes, 1,886 phenotypes, and 2,603 gene-phenotype associations were reserved. Theoretically, 1 was defined as the weighted score for each phenotype-gene association.

### Phenotype-metabolite association network

First of all, phenotype-metabolite associations were obtained from the HMDB. Then, 664 associations between 388 metabolites and 149 pheno-types were reserved after filtration. The weighted score was determined as 1 for each phenotypemetabolite interaction.

### Establishment of a heterogeneous network

With the goal of prioritizing the potential metabolites, the above six composite networks mentioned were integrated into a weighted composite network, namely a heterogeneous network. The details were described in then literature (16).

### Prioritization of candidate metabolite based on the heterogeneous network

We firstly extracted the known disease metabolites from the Human Metabolome Database (HMDB) (17) which collected the specific information of small-molecule metabolites of human and the disease-related phenotypes described in the OMIM. The known ALI-associated genes were retrieved from the Morbid Map file of Online Mendelian Inheritance in Man (OMIM) database (18). After obtaining these data, we mapped these seed genes and known disease metabolites to the heterogeneous network.

To prioritize the candidate metabolites from the heterogeneous network, we utilized RWR method to expand to the heterogeneous network (19). In theory, RWR prioritized potential metabolites according to the proximity of every candidate metabolite to the ALI-related seed genes within the network and simulating a random walker starting with the seed nodes. At every step, the walker moved from the current node to its direct neighbors with probability 1-β or returned to the seed nodes with probability β. We then ranked these candidate metabolites using distance proximity. Based on the distance proximity, the top 50 metabolites with the greatest scores were identified and defined as the ALI-prioritized metabolites.

Using the top 50 metabolites and the heterogeneous network, we detected several genes interacted with the top 50 metabolites, and then we extracted the top 100 co-expressed genes relying on the score distribution.

The sub-network about the top 50 metabolites obtained from the composite network, and the co-expressed network were all constructed. Of note, in order to uncover significant metabolites involved in ALI, we implemented degree analyses for these two networks.

## Results

### DEGs identification and establishment of a integrated multi-omics composite network

Firstly, DEGs were identified which were used to further establish the gene-gene network. When the criteria was set as the FDR < 0.05, a total of 560 DEGs were screened out between the two groups.

Then, an integrated multi-omics composite network was built through merging 3 kinds of data (metabolome, genome, and phenome), and 6 kinds of interactions (metabolite-metabolite, gene-gene, phenotype-phenotype, gene-metabolite, phenotype-gene, as well as pheno-type-metabolite). Overall 9363 nodes and 10,226,148 edges were included in this composite network.

### Prioritizing the ALI-related metabolites

Totally, 6 ALI-related risk genes are stored in the OMIM database, including ACE2, TGFB1, TLR2, TLR4, ANGPT2, AGTR1, which were extracted as seed genes from the OMIM database. The OMIM ID for ALI is 178500, and no known disease metabolites about ALI in HMDB. In our analysis, the whole metabolome as candidates, the phenotype of ALI, and the 6 disease-related genes of ALI were used as seeds. To declare the intrinsic mode of this method, the metabolites within the weighted composite network were ranked in descending order relying on the interaction scores, and the top 50 metabolites with the highest scores were extracted. The top 10 metabolites was shown in Table 1.

**Table 1:** List of the top 10 metabolites

***Rank***	***Metabolite CID***	***Metabolite Name***	***Score***
1	24139	CHITIN	0.001229
2	444795	Tretinoin (TN)	0.001227
3	923	sodium ion	0.000964
4	27476	1-Methyladenosine	0.000939
5	2662	Celebrex (TN)	0.000902
6	5280360	Cervidil (TN)	0.000747
7	5994	Prometrium (TN)	0.00068
8	79014	D-glyceraldehyde	0.000657
9	6022	Adenosine 5′-diphosphate	0.000589
10	5757	Estraderm (TN)	0.000581

**Fig. 1:**
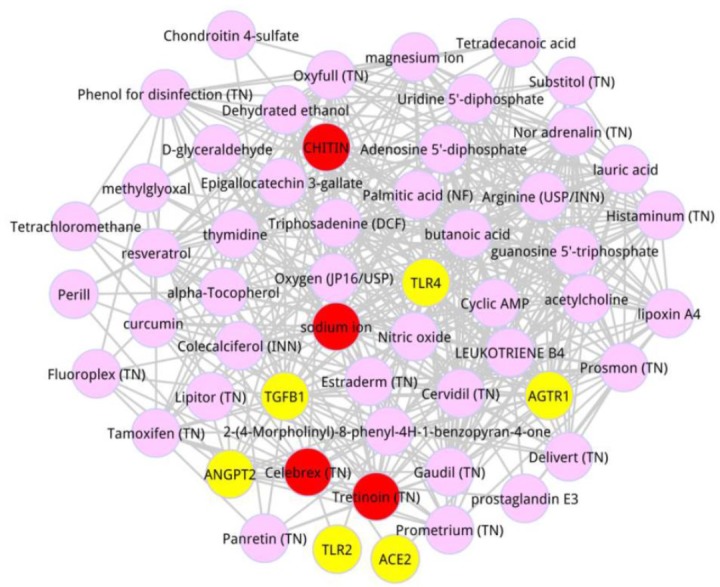
Composite sub-network of the top 50 metabolites and the 6 seed genes. Yellow nodes were the ALI-related seed genes obtained from the OMIM database. Pink nodes denoted the metabolites, and red ones were on behalf of the metabolites with the distance proximity > 0.009

There were 4 metabolites with the scores > 0.009, including CHITIN (score = 0.001229), Tretinoin (score = 0.001227), sodium ion (score = 0.000964), and Celebrex (score = 0.000902). A subnetwork for the top 50 metabolites was extracted from the whole composite network, as listed in Fig. 1. Adenosine 5′-diphosphate had the highest degree of 29, and Triphosadenine owned the higher degree of 27.

**Fig. 2:**
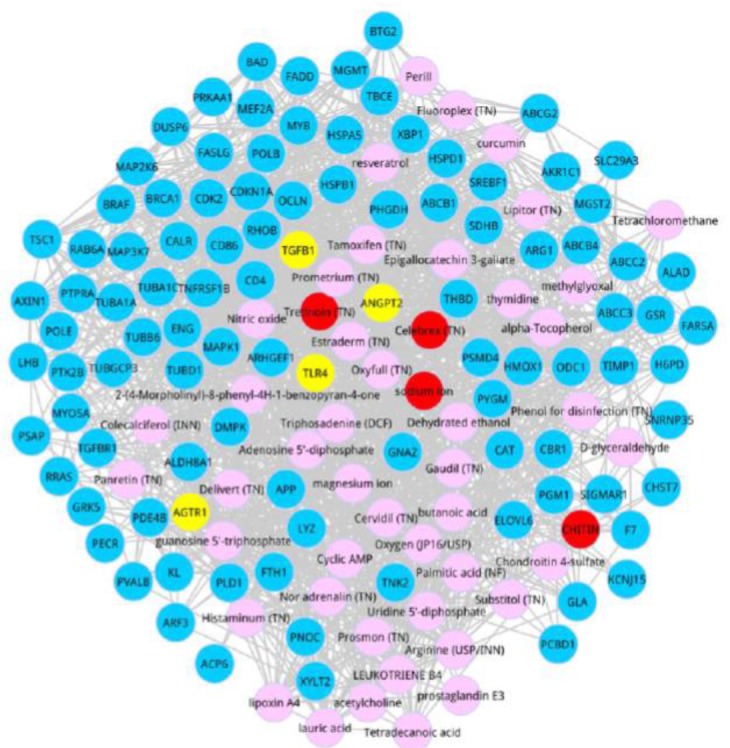
Co-expressed network. Blue nodes were the top 100 co-expressed genes. Yellow nodes devoted the members of ALI-related seed genes. Pink nodes were the metabolites, and Red ones were the metabolites with the distance proximity > 0.009

Next, we detected the co-expressed genes interacted with the top 50 metabolites based on the score ranking. On the basis of setting criteria previously, the top 100 co-expressed genes were identified, and the co-expressed network of the top 100 genes was exhibited in Fig. 2. After degree analysis for the co-expressed network, there were 5 metabolites with the degree not less than 55, including Adenosine 5′-diphosphate (degree = 65), triphosadenine (degree = 64), Estraderm (degree = 59), tretinoin (degree = 55), and magnesium ion (degree = 55). Significantly, tretinoin was the member of the top 5 metabolites.

## Discussion

Although remarkable progress has been made in the management of ALI induced by sepsis, the prevention and early diagnosis are crucially important. It is helpful for us to uncover diagnostic biomarkers for ALI. Through the literatures, finding disease-related metabolites is a key step for increasing clinical diagnosis (10, 20). “Omics” data (including metabolic, phenomic, and genomic), will provide available information to prioritize the disease-related candidate metabolites. More significantly, one disease is frequently caused by the interactions among functionally related genes and metabolites that organize into a complicated network. Naturally, combining metabolite, gene, and phenotype data is an effective method to build a composite network to further identify disease-risk metabolites, and this integration approach can offer comprehensive and accurate information (21, 22). Few efforts have been done to reveal the possible involvement of metabolisms in ALI, thus, we aimed to detect the candidate metabolites in ALI to further explore the underlying mechanisms of ALI.

In our study, a metabolite adenosine 5′-diphosphate had the highest degree in the subnetwork for the top 50 metabolites and the co-expressed network. The primary roles of poly adenosine diphosphate-ribose polymerase (PARP) are to repair DNA, sense DNA damage, and maintain genomic stability (23). Further, up-regulation of PARP might be detrimental through removing cellular ATP stores, thereby leading to cell dysfunction and death (24). Adenosine 5′-diphosphate ribose synthetase exerts key functions in ALI in pigs (25). Remarkably, suppression of PARP has been implicated to attenuate the lung injury induced by ventilator (26). Accordingly, adenosine 5′-diphosphate might play crucial roles in the progression of ALI via regulating the cellular suicide mechanism (27). Another metabolite triphosadenine owned the higher degree in the subnetwork for the top 50 metabolites and the co-expressed network. A former study has implicated that triphosadenine can increase the expression level of ROS (28). Furthermore, increased production of ROS is associated with the increased oxidative stress (29). Oxidant stress is implicated to be a major contributor in the ALI progression (30). Additionally, the protective mechanisms of several drugs (for example, rutin, quercetin and trillin) for lipopolysaccharide-induced ALI is through suppression of oxidative stress (31–33). Thus, we speculate that the metabolite triphosadenine is closely related to the pathology of ALI. Moreover, the metabolite tretinoin had the higher degree in this analysis.

As reported, tretinoin is the main active form of vitamin A in the organism, which plays a variety of roles in regulating cell growth, differentiation, modulating inflammatory as well as immune response, and repairing cell injury (34). More recently, the tretinoin was implicated to influence branching morphogenesis of lung through the interactions with several genes participating in lung development (35).

Treatment with tretinoin may improve the alveolar structure, and reduce alveolar septal fibrosis in preterm infants (36). Oxidative stress is suggested to play a key role in the development of ALI. It was shown that tretinoin, as the antioxidants, was believed to protect the lung against damage induced by oxygen free radicals (37). Consequently, we believe that tretinoin might be related to the progression of ALI.

## Conclusion

Chip data from patients with ALI induced by sepsis were compared with those from sepsis patients to detect candidate metabolites which may play a role in ALI. Relevant metabolites like Adenosine 5′-diphosphate, triphosadenine, and tretinoin might be potential biomarkers for diagnosis and therapy of ALI. However, further experiments will be done to confirm our findings.

## Ethical considerations

Ethical issues (Including plagiarism, informed consent, misconduct, data fabrication and/or falsification, double publication and/or submission, redundancy, etc.) have been completely observed by the authors.
